# Exploring predictors of dysphagia in survivors of head and neck cancer: A cross-sectional study

**DOI:** 10.1007/s00520-024-08358-w

**Published:** 2024-02-17

**Authors:** María Dolores López-Fernández, Carolina Fernández-Lao, Alba María Ruíz-Martínez, Miguel Ángel Fernández-Gualda, Mario Lozano-Lozano, Lucía Ortiz-Comino, Noelia Galiano-Castillo

**Affiliations:** 1 UGC Medicina Física y Rehabilitación, Hospital de Neurotraumatología y Rehabilitación, Hospital Universitario Virgen de Las Nieves, Instituto de Investigación Biosanitaria Ibs, Granada, Spain; 2 Biomedical Group (BIO277), Department of Physical Therapy, Health Sciences Faculty, University of Granada, Sport and Health Research Center (IMUDs), Instituto de Investigación Biosanitaria Ibs, Granada, Spain; 3 Department of Radiation Oncology, Hospital Universitario Vírgen de Las Nieves, Instituto de Investigación Biosanitaria Ibs, Granada, Spain; 4 Biomedical Group (BIO277), Department of Physical Therapy, Health Sciences Faculty, University of Granada, Instituto de Investigación Biosanitaria Ibs, Granada, Spain; 5 Biomedical Group (BIO277), Department of Physical Therapy, Health Sciences Faculty (Melilla), University of Granada, Sport and Health Research Center (IMUDs), Instituto de Investigación Biosanitaria Ibs, Granada, Spain

**Keywords:** Head and Neck Neoplasms, Deglutition Disorders, Trismus, Cough, Sleep Quality

## Abstract

**Purpose:**

To evaluate the prevalence of dysphagia in survivors of head and neck cancer (sHNC) and to identify the predictors contributing to the development of dysphagia.

**Methods:**

We enrolled 62 sHNC in a cross-sectional study to check the prevalence of dysphagia in sHNC and to evaluate which factors were influencing the presence of this side effect. Besides dysphagia, sociodemographic and clinical characteristics, oral symptoms, maximal mouth opening (MMO), sleep quality and physical condition were evaluated, and a linear regression analysis was performed to verify which of these outcomes impact dysphagia.

**Results:**

Among all the sHNC, 85.5% presented dysphagia. The linear regression analysis confirmed that 44.9% of the variance in dysphagia was determined by coughing, MMO and sleep quality, being MMO the most powerful predictor, followed by coughing and sleep quality.

**Conclusion:**

Dysphagia affected the great majority of sHNC. Moreover, symptoms as coughing, reduced MMO and sleep disorders may act as predictors contributing to the development of dysphagia. Our results emphasize the importance of an early and proper identification of the symptoms as well as an adequate treatment strategy to address the cluster of symptoms that sHNC undergo.

**Supplementary Information:**

The online version contains supplementary material available at 10.1007/s00520-024-08358-w.

## Introduction

Dysphagia, defined as limited swallowing function, affects more than half (54.9%) of survivors of head and neck cancer (sHNC) [1]. Beyond the physical limitations of swallowing, dysphagia poses a substantial risk of complications that can significantly affect the overall health and well-being of sHNC. The key challenges associated with dysphagia include medication nonadherence [2], malnutrition [3], dehydration [2], and an increased susceptibility to pneumonia [4]. Dealing with dysphagia and its associated complications can also evoke feelings of depression and anxiety, while eating and communication disorders may lead to social isolation [5]. Thus, the impact of dysphagia on sHNC extends far beyond the immediate physical consequences, affecting their overall quality of life (QoL).

Consequently, dysphagia should be considered a ‘vital sign’ beyond swallowing dysfunction. Head and neck cancer (HNC) guidelines recommend the use of multidisciplinary teams to monitor and optimize dysphagia outcomes and intervene when necessary [2]. Despite this recommendation, health care professionals lack the tools to identify patients who are at risk of suffering dysphagia due to a lack of understanding of which potential outcomes may influence this troubling side effect of cancer and its treatment.

On the one hand, relationships among trismus (i.e., impaired mouth opening), QoL, and dysphagia in large populations of sHNC have been previously shown [6]. On the other hand, Petersson and colleagues [7] investigated whether radiation-derived dysphagia could be predicted by tumour and patient characteristics such as feeding tube use, weight factors, trismus, and saliva secretion in sHNC. Furthermore, Ortiz-Comino [8] and colleagues found that pain perceived in the cervical and shoulder regions, perception of physical fitness, and fatigue were predictors of QoL among sHNC. Finally, Pezdirec and colleagues [9] found a significant relationship between dysphagia and some of the treatment-derived side effects (impaired mouth opening, sticky saliva, and persistent cough, among others), radiotherapy (RT), and symptoms of gastroesophageal reflux, but after multiple regression modelling, only persistent cough remained significant. However, the interplay between dysphagia and outcomes related to oral symptoms and mouth opening, sleep quality, physical condition and treatment modality has not yet been characterized in this population.

Sleep disturbances have been found to be prevalent in sHNC, and these can have a significant impact on swallowing function [10]. Moreover, the relationship between sleep disorders and dysphagia resulting from HNC becomes even more significant when considering the marked sequelae of xerostomia and sticky saliva following treatment for oropharyngeal cancers [11]. Disrupted sleep patterns and poor sleep quality can lead to fatigue, decreased muscle coordination, and weakened muscle tone in the oropharyngeal area, all of which can exacerbate swallowing difficulties [12]. Additionally, sleep disorders may contribute to inflammation and tissue damage in the upper aerodigestive tract, further compromising the swallowing process [13]. Therefore, addressing and managing sleep disorders in sHNC may play a crucial role in improving dysphagia outcomes and overall QoL.

Likewise, patients with dysphagia may not be able to swallow certain foods or drinks, which can lead to a restricted diet; the resulting poor nutritional status may worsen when the swallowing-related muscles are affected by sarcopenia, aggravating dysphagia. Furthermore, the loss of skeletal muscle mass (SMM) and physical function that accompany sarcopenia leads to a vicious cycle, further worsening the swallowing ability of sHNC [14].

Prior research involving sHNC has shown a direct relationship between a more complex treatment (higher radiation doses or concurrent chemotherapy-radiotherapy) and greater dysphagia [15]; In contrast, Jia and colleagues did not find a significant relationship between dysphagia and treatment modality (surgery and/or adjuvant treatment) [6].

To date, no studies have evaluated the complex relationships among dysphagia, oral symptoms, maximal mouth opening (MMO), sleep quality, and physical condition. The purpose of this study was to evaluate the prevalence of dysphagia in sHNC and to identify the predictors contributing to the development of dysphagia by analysing whether a 10-question symptom-specific measure of dysphagia symptoms [(Eating Assesment Tool (EAT-10)] correlated with a worsening in oral symptoms, MMO, or sleep quality; an increased risk of physical deconditioning; and a more complex treatment modality in sHNC. The results of this study may emphasize the importance of recognizing predictors of dysphagia and reinforce the importance of early and accurate identification and treatment in this population.

## Methods

### Study design and setting

This cross-sectional study followed the STROBE statement checklist recommendations [16] (Online resource) and was approved by the Biomedical Investigation Ethics Committee, Granada, Spain (0045-N-16 and 1552-N-18) and performed according to the Declaration of Helsinki. Participants were recruited between 2018 and 2022 at the Virgen de las Nieves University Hospital and San Cecilio University Hospital (Granada, Spain). The assessments were carried out at the facilities of the research group Cuídate (Physical Therapy Department, University of Granada, Spain).

### Participants

sHNC were enrolled if they met the following inclusion criteria: diagnosis of a cancer located in the upper aerodigestive tract, 18 years of age or older, oncological treatment completed, in complete remission, and Spanish speaking; the exclusion criteria were cognitive or social characteristics that would hinder understanding and completing the assessment, presence of metastases, and refusal to participate. All participants signed an informed consent statement before participation.

### Measures

Once sHNC had been recruited, all measurements were obtained by the research staff in a single visit.

#### Sociodemographic and clinical variables

Sociodemographic characteristics were obtained by standard questions and included age, gender, alcohol and tobacco consumption, civil status and education level. Clinical variables were collected from the medical records and included primary tumour location, tumour stage at diagnosis, time since diagnosis and treatment modality.

#### Dysphagia

The perception of swallowing dysfunction was measured with the EAT-10. This is a validated symptom-specific self-report questionnaire consisting of 10 items scored on a 0–4 scale (0 = no impairment; 4 = a severe problem). The total score ranges from 0 to 40, with ≥ 3 points considered indicative of dysphagia (Cronbach’s alpha = 0.96) [17].

#### Oral symptoms: coughing and sticky saliva

Coughing and sticky saliva were evaluated with the European Organization for Research and Treatment of Cancer Quality of Life specific module for head and neck (EORTC QLQ-H&N35) [18]. We used these two symptom items related to coughing and sticky saliva perception scored on a 4-point Likert scale from 1 (not at all) to 4 (very much); a higher score indicates a higher level of symptomatology. This questionnaire has been previously validated in the HNC population (Cronbach’s alpha > 0.70) [19].

These two items were chosen because they were relevant to the aim of this study according to previous studies that have highlighted unmet needs in the HNC population related to QoL after oncological treatment [11].

#### Maximal mouth opening

MMO was measured in millimetres (mm) using a sliding calliper. sHNC who used a dental prosthesis were instructed to wear their prosthesis during the measurements. Patients were asked to stand and to open their mouth as wide as possible, and then the distance between the upper and lower central incisors (of their own dentition or prosthesis) was measured. An MMO of ≤ 35 mm is used as the cut-off point for trismus in the HNC population [20] and has good reliability (intraclass correlation coefficient [ICC] = 0.95–0.96) [21].

#### Sleep quality

Sleep quality was evaluated using the self-reported Pittsburgh Sleep Quality Index (PSQI). The PSQI global score ranges from 0 to 21 and discriminates good sleepers from poor sleepers, with a higher score indicating worse sleep quality. A global score ≥ 5 points indicates poor sleep quality and has shown consistency and validity in cancer research (Cronbach’s alpha = 0.77–0.81) [22].

#### Physical condition: physical functioning and skeletal muscle mass

Physical functioning was evaluated with a subscale of the European Organization for Research and Treatment of Cancer Quality of Life Core-30 version 3.0 (EORTC QLQ-C30). This subscale is composed of five items related to difficulties in daily activities. The items are scored on a 4-point Likert scale from 1 (not at all) to 4 (very much), with higher scores indicating higher levels of physical functioning. This questionnaire has been validated in the HNC population (Cronbach’s alpha > 0.70) [19].

We hypothesized that this physical functioning domain should be part of the comprehensive assessment, as HNC patients present QoL impairment with regard to physical limitations [11].

A bioelectrical impedance analysis (BIA) was performed using InBody 720 (InBody Corp. Seoul, Korea) to evaluate SMM (kg). This machine uses a tetrapolar eight-point tactile electrode system, where participants must stand barefoot on the feet electrodes and with their hands gripping the hand electrodes, with the arms abducted along the trunk. In addition, they were instructed to not alter their normal activity and to not eat just before the test. The BIA method is a valid tool for estimating SMM [23].

### Statistical analyses

Continuous variables are presented as the mean ± standard deviation (95% confidence interval), and categorical data are represented as frequencies and percentages. The Kolmogorov‒Smirnov test was performed to assess whether the variables were normally distributed.

To minimize the potential bias arising from missing data, multiple imputation was employed for missing data. We assumed that the data were missing at random conditional on measured characteristics, and excluding participants with any missing data can introduce bias and decrease statistical power. A total of 25 different imputed databases were generated, which included multiple estimates for the missing data. The pooled data were subsequently utilized for analysis [24]. Then, a bivariate correlation analysis was performed to examine the correlations among dysphagia, coughing, sticky saliva, MMO, sleep quality, physical functioning, SMM and treatment modality using the Pearson or Spearman correlation coefficient depending on the normality of the variables. The correlation coefficients were categorized according to the Cohen criteria (> 0.5, large; 0.5–0.3, moderate; < 0.3–0.1, small; and < 0.1, insubstantial) [25]. Subsequently, a partial correlation analysis was performed adjusting for three sociodemographic and clinical covariates one by one (age, tumour stage and time since diagnosis), and semipartial correlations were also obtained to examine the specific contribution of each independent variable.

The variables significantly correlated with dysphagia and with a correlation coefficient ≤ 0.7 between each other were entered in a linear regression analysis (stepwise method) to determine the association between potential predictors (independent variables) and dysphagia (dependent variable). Multicollinearity between independent variables was assessed using the variance inflation factor (VIF) and tolerance; if the VIF and tolerance were > 10 and < 0.2, respectively, multicollinearity existed [26]. Furthermore, the regression model was adjusted for age, tumour stage and time since diagnosis. Finally, based on clinical and statistical criteria, the mediation effect of sticky saliva, MMO, and sleep quality on the association between coughing, MMO, sleep quality, and treatment modality with dysphagia was assessed with the PROCESS macro for IBM SPSS Statistics. Statistical significance was defined as p < 0.05. All statistical analyses were performed using the software IBM SPSS Statistics version 28.0 (IBM, Armonk, NY), and the graph was made using RStudio (R 4.3.1 software).

## Results

### Study population

A total of 62 sHNC with a mean age of 60.69 ± 11.08 years participated in the study, of whom 69.4% (n = 43) were men. The most common primary tumour location was the oropharynx and oral cavity (37.1%, n = 23), and the most common treatment modality was a combination of RT, surgery and chemotherapy (41.9%, n = 26), followed by RT plus chemotherapy (29%, n = 18) and RT plus surgery (24.2%, n = 15). Among all the sHNC, 85.5% (n = 53) presented dysphagia (EAT-10 ≥ 3). Table 1 details all the sociodemographic and clinical characteristics of the sample.

**Table 1:** Sociodemographic and clinical characteristics of the study population

	n = 62
Age (years), mean (± SD); CI 95%	60.69 (11.08); 57.88 – 63.51
Gender, n (%)	
Male	43 (69.4)
Female	19 (30.6)
Alcohol consumption^a^, n (%)	
Yes No Previous	28 (45.2) 26 (41.9) 7 (11.3)
Tobacco consumption, n (%)	
Yes No Ex-smoker	7 (11.3) 22 (35.5) 33 (53.2)
Civil status, n (%)	
Single/divorced Married Widowed	15 (24.2) 41 (66.1) 6 (9.7)
Education level, n (%)	
Basic Medium High No studies	23 (37.1) 17 (27.4) 15 (24.2) 7 (11.3)
Primary tumour location^b^, n (%)	
Nasopharynx and cavum Oropharynx and oral cavity Tongue Major salivary glands Larynx and hypopharynx Others*	9 (14.5) 23 (37.1) 2 (3.2) 4 (6.5) 13 (21) 3 (4.8)
Tumour stage^c^, n (%)	
I II III IIIA IV IVA IVB IVC	5 (8.1) 7 (11.3) 7 (11.3) 7 (11.3) 10 (16.1) 14 (22.6) 5 (8.1) 2 (3.2)
Time since diagnosis (months), mean (± SD); CI 95%	32.60 (20.86); 27.30 – 37.89
Treatment modality, n (%)	
RT RT + surgery RT + chemotherapy RT + surgery + chemotherapy	3 (4.8) 15 (24.2) 18 (29) 26 (41.9)
Outcomes, mean (± SD); CI 95%	
Dysphagia (EAT-10)	15.42 (11.01); 12.63 – 18.22
Oral symptoms	
Coughing (EORTC QLQ-H&N35) Sticky saliva (EORTC QLQ-H&N35) MMO (mm) Sleep quality (PSQI)	27.87 (30.54); 20.05 – 35.69 59.56 (37.07); 50.07 – 69.06 33.85 (11.30); 30.98 – 36.72 9.53 (4.91); 8.29 – 10.78
Physical condition	
Physical functioning (EORTC QLQ-C30) SMM (kg)	82.40 (20.15); 77.24 – 87.56 29.39 (6.34); 27.75 – 31.03

CI: confidence interval; EAT-10: Eating Assessment Tool-10; EORTC QLQ-C30: European Organization for Research and Treatment of Cancer Quality of Life Core-30; EORTC QLQ-H&N35: European Organization for Research and Treatment of Cancer Quality of Life specific module for head and neck; kg: kilograms; mm: millimetres; MMO: maximal mouth opening; PSQI: Pittsburgh Sleep Quality Index; RT: radiotherapy; SD: standard deviation; SMM: skeletal muscle mass

^*^: maxillary sinus, cervical lymph nodes; a: n = 61, b: n = 54, c: n = 57

### Association between dysphagia and oral symptoms, MMO, sleep quality, physical condition, and treatment modality

**Fig. 1:**
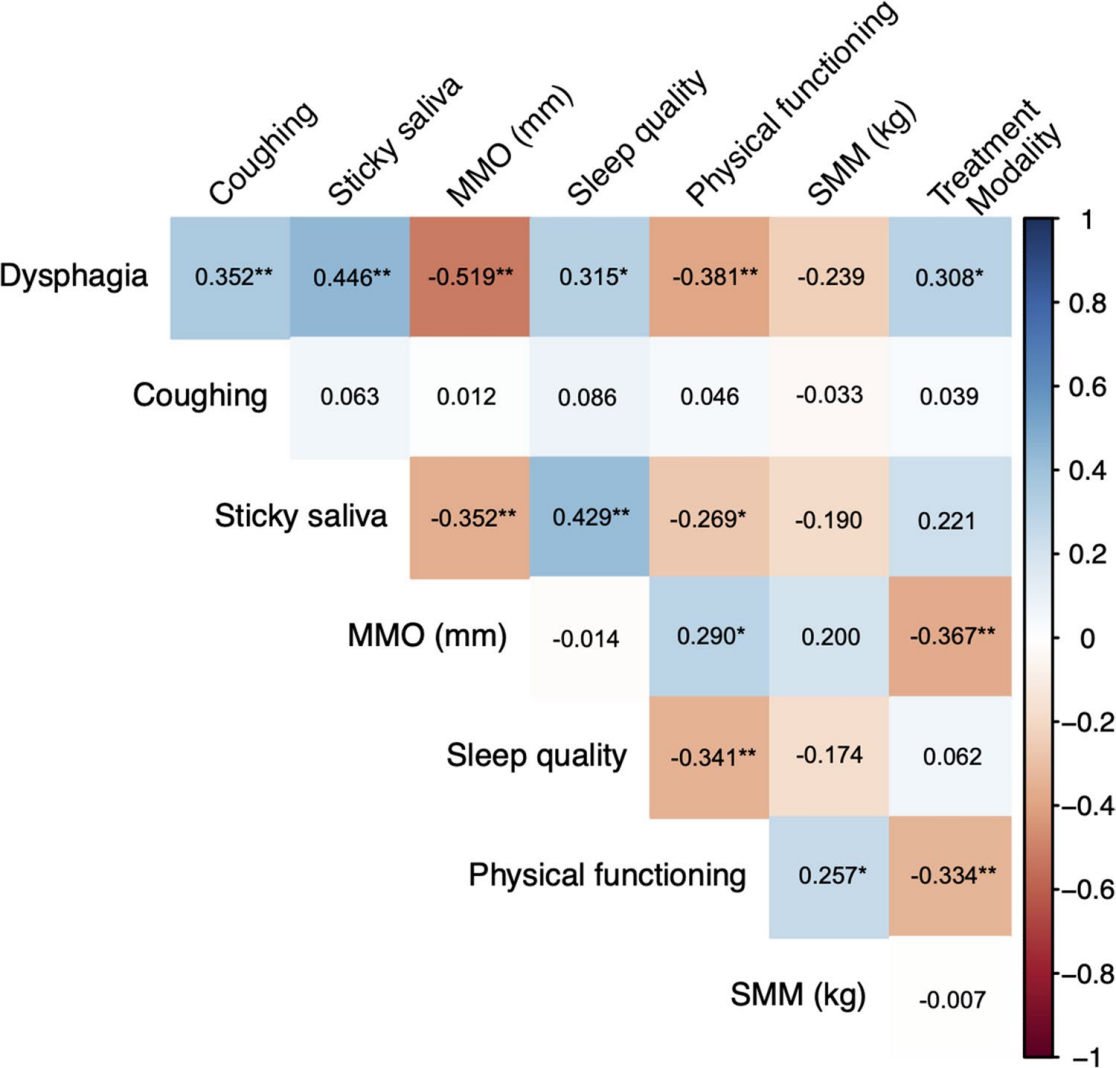
Correlation matrix between dysphagia, coughing, sticky saliva, maximal mouth opening (MMO), sleep quality, physical functioning, skeletal muscle mass (SMM) and treatment modality. The blue and red colour gradient represents the strength of the correlation coefficient (Pearson for all variables except Spearman for treatment variable). *p < 0.05; **p < 0.01

Figure 1 shows that a higher level of dysphagia was correlated with worse coughing (r = 0.352, *p* = 0.005), worse sticky saliva (r = 0.446, *p* < 0.001), worse sleep quality (r = 0.315, *p* = 0.013), worse physical functioning (r = -0.381, p = 0.002) and more complex treatment modality (r = 0.308, *p* = 0.015). Moreover, a higher level of dysphagia was correlated with a lower MMO (r = -0.519, *p* < 0.001). No significant correlation between dysphagia and SMM was noted (r = -0.239, p = 0.066).

### Partial and semipartial correlations

After adjusting for age, higher dysphagia correlated with lower SMM (r = -0.266, p = 0.043). After adjusting for tumour stage, higher dysphagia correlated with lower SMM (r = -0.289; p = 0.036), and treatment modality lost its significant correlation with dysphagia (r = 0.258, p = 0.062). The statistically significant associations with all other independent variables remained the same after adjusting for both age and tumour stage. All the associations persisted after adjustment for time since diagnosis.

Semipartial correlations for each predictor variable showed that MMO explained 27.04% of dysphagia variance (r = -0.520), coughing explained 11.08% (r = 0.333) and sleep quality explained 7.95% (r = 0.282).

### Multiple regression model

Although coughing, sticky saliva, MMO, sleep quality, physical functioning and treatment modality were significantly correlated with dysphagia (p < 0.05), linear regression analysis confirmed that 44.9% of the variance in dysphagia was determined by coughing, MMO and sleep quality (adjusted r^2^ = 44.9%, F = 17.284, *p* < 0.001). MMO was the most powerful predictor for dysphagia, followed by coughing and sleep quality. For every unit decrease in MMO measurement, the expected value of EAT-10 increased by 0.520 units; for every unit increase in coughing (EORTC QLQ-H&N35), the expected value of EAT-10 increased by 0.334 units; and for every unit increase in PSQI global score, the expected value of EAT-10 increased by 0.283 units (Table 2).

**Table 2:** Multiple linear regression analysis (stepwise) to determine predictors of dysphagia (r^2^ = 44.9%)

Independent variables	β	T	p
Maximal mouth opening (mm)	- 0.520	- 5.421	< 0.001
Coughing (EORTC QLQ-H&N35)	0.334	3.474	< 0.001
Sleep quality (PSQI)	0.283	2.937	0.005

EORTC QLQ-H&N35: European Organization for Research and Treatment of Cancer Quality of Life specific module for head and neck; mm: millimetres; PSQI: Pittsburgh Sleep Quality Index

The multicollinearity analysis in the regression model showed no correlation among MMO (VIF = 1.000, tolerance = 1.000) coughing (VIF = 1.008, tolerance = 0.992) and sleep quality (VIF = 1.008, tolerance = 0.993).

The regression model was not modified after adjusting for age, tumour stage and time since diagnosis.

**Fig. 2:**
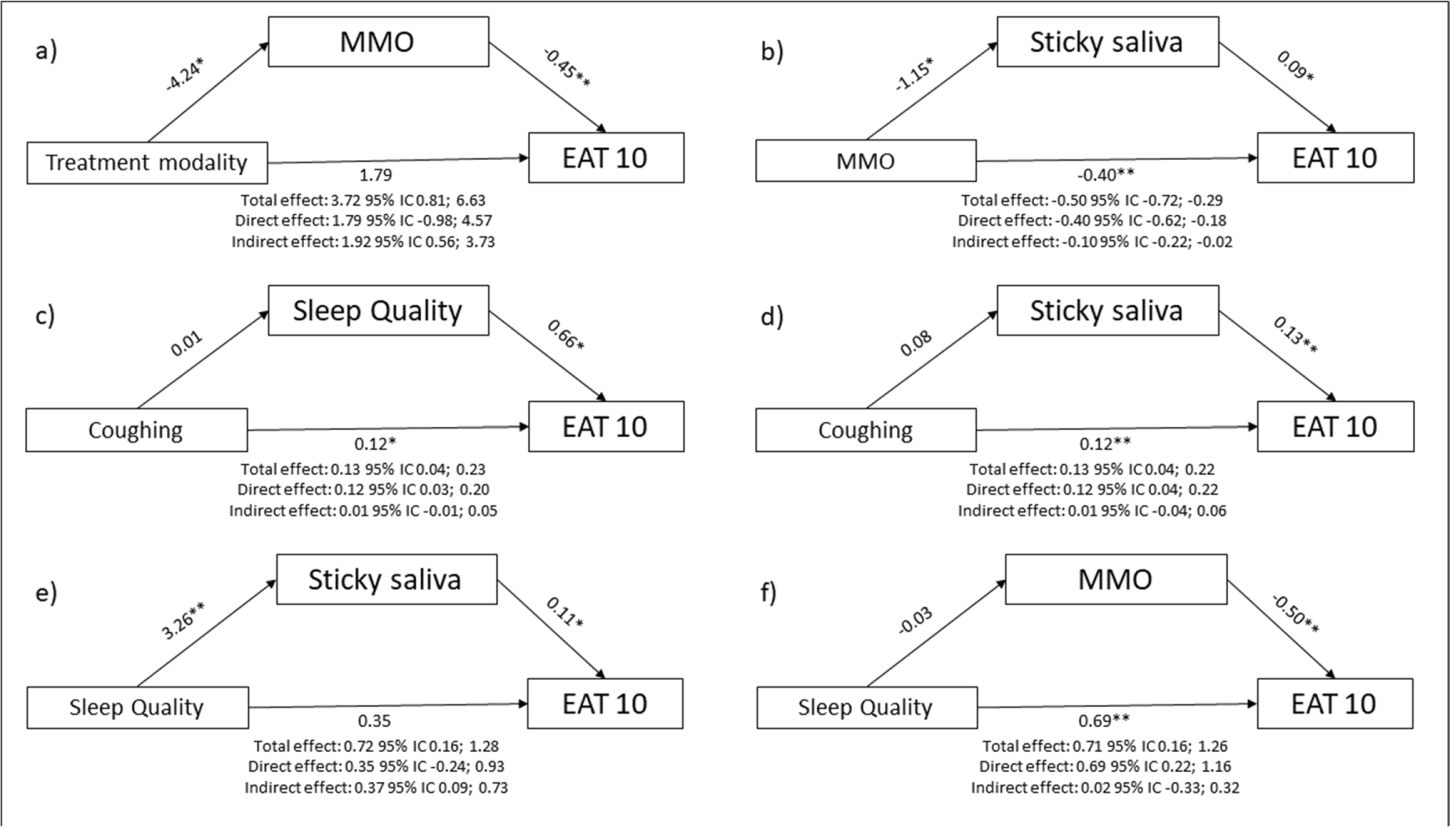
Mediation effects of maximal mouth opening (MMO), sticky saliva and sleep quality on the association between treatment modality, maximal mouth opening, coughing and sleep quality with dysphagia (EAT10). EAT-10: Eating Assessment Tool. *p < 0.05, **p < 0.001

Figure 2 represents the mediating effects of MMO, sticky saliva and sleep quality on the association between treatment modality, MMO, coughing and sleep quality, as appropriate. As shown, the influence of treatment modality on dysphagia was partially explained through a reduction in MMO (indirect effect ranging from 0.56 to 3.73; p < 0.05) (Fig. 2_a). A similar significant indirect effect of sticky saliva was found in the relationship between MMO and dysphagia (-0.22 to -0.02; p < 0.05) (Fig. 2_b) and between sleep quality and dysphagia (0.09 to 0.73; p < 0.05) (Fig. 2_e).

## Discussion

The goal of this study was to examine potential predictors of dysphagia in sHNC and the associations between these outcomes. According to the Cohen criteria, a substantial association between dysphagia and MMO was found, whereas moderate associations were found between dysphagia and coughing, sticky saliva, sleep quality, physical functioning, and treatment modality; only three of these outcomes (MMO, coughing and sleep quality) could partly explain this swallowing dysfunction. Dysphagia is one of the main RT-induced side effects, among other acute and chronic side effects of oncological treatment due to the traumatizing impact on the tissues surrounding the upper aerodigestive tract [1], especially when surgery, RT, and chemotherapy coexist [27].

Our study population was 60.69 years old on average and had been diagnosed with cancer for a mean of 32.6 months, and the great majority (85.5%; 53 participants) presented dysphagia as detected by the EAT-10; results that concord with a previous cross-sectional study [28]. Moreover, our study population presented oral symptoms such as coughing and sticky saliva (items extracted from the EORTC QLQ-H&N35 questionnaire). These symptoms, added to the sensory deterioration due to oncological treatment may induce an inefficient cough reflex and thus increase the risk of dysphagia [29]. Consistent with our results, Nguyen and colleagues [30] found that only 40% of patients with HNC with dysphagia have an effective cough response to aspirated material, and, as described by Beetz and colleagues, [31] 43% of patients with HNC reported moderate to severe sticky saliva at 6 months after treatment with primary chemoradiotherapy. Another symptom presented by our population was trismus, a side effect commonly suffered by sHNC [32]; poor sleep quality occurs in approximately one-third of patients with HNC after treatment [33] and continues in the long term [34]. Finally, the studied population also presented low levels of physical functioning (evaluated through the related subscale from the EORTC QLQ-C30) and poor SMM [35], which is considered common in survivors with cancer and specifically in sHNC [36]. Decreasing weight and SMM are usually linked to malnutrition among sHNC, even in patients who are able to maintain a normal weight, which could be related to severe dysphagia [7]. Moreover, those who perform more leisure-time exercise have a lower risk of dysphagia [37], as leisure activities are usually associated with higher tongue pressure, suggesting that interacting with others may be one way of maintaining oral function [38].

The association between dysphagia and MMO was the strongest in our study. Impaired MMO is usually associated with radiation fibrosis syndrome [39]. In the HNC population, the prevalence of trismus ranges from 6 to 79% [40]. This symptom is one of the most common symptoms presented in sHNC, affecting up to 40% of patients after oncological treatment [40]. As mouth opening is crucial to the oral phase of swallowing, its decrease could indeed induce dysphagia [27]; in addition, limited MMO may cause difficulties in proper food mastication, which is linked to aspiration problems due to compromised airway clearance with poor bolus organization [41].

To date, no studies have evaluated the association between dysphagia and sleep quality, but if symptoms such as coughing and sticky saliva occur at night, it could be possible that sleep quality may also deteriorate. Considering the mediation effect of sticky saliva on the association between sleep quality and dysphagia, it seems logical explore further the aforementioned mediation in poor sleepers (see Fig. 2_e).

Finally, SMM only correlated negatively with dysphagia when adjusting for either age or tumour stage in this study, a result concordant with previous studies that associate ageing processes with the loss of SMM or even sarcopenia [42]; similarly, a greater tumour stage is usually associated with more aggressive treatments that could worsen SMM, affecting the swallowing process. Although there is some disagreement on the effects of the treatment modality on the perception of dysphagia, our results demonstrate a moderate association between dysphagia and treatment modality, but the latter was not predictor contributing the development of dysphagia after our multiple regression modelling. This could be due to the true association between treatment modality with dysphagia is mediated significantly by MMO (see Fig. 2_a). We included this variable in our study because oncological treatment has been shown to induce secondary side effects over the cranio-cervical region [43], which could secondarily worsen the swallowing process. Finally, after adjusting for tumour stage, treatment modality did not correlate with dysphagia.

Moreover, our results revealed that MMO, coughing, and sleep disorders explains 44.9% of the variability of the overall problem, acting as predictors of dysphagia presented by sHNC, with MMO being the most powerful predictor in our model. All predictors found in this study were independently associated with dysphagia, as no collinearity was found between them when analysed.

As stated previously, MMO impairments are widely present in sHNC [40], and their decrease could directly be associated with dysphagia [27]. Similarly, cough could be related to hyposensitivity of the larynx, producing a microaspiration in the trachea after surgery/irradiation. On the other hand, gastroesophageal reflux is known to be associated with obstructive apnoea [44], and this fact could play an important role in the development of sleep disorders in our population [45]. To date, no studies have stated that sleep quality perception is a predictor of dysphagia in sHNC, but the loss of restful sleep could induce sHNC to have a higher perception of fatigue during the day [46], and this fact could increase the risk of perceiving dysphagia.

Identifying independent factors associated with dysphagia is crucial to guide health care professionals in the identification of patients at higher risk of dysphagia, so that interventions focused on these outcomes (i.e., MMO, coughing and sleep quality) may prevent their worsening and thus the risk of aspiration, as previously mentioned.

The study limitations are as follows. First, the heterogeneity of the tumour locations and the different stages of the disease and treatment modalities used in the studied population. It would be desirable to increase the sample size to conduct a subgroup analysis to determine the stability of the cluster in different tumour locations or treatment modalities. Moreover, due to the cross-sectional design, we did not perform a follow-up assessment of the patients, which would have been important to analyse changes in the outcomes evaluated at different time points. Longitudinal research with the inclusion of a follow-up registry will enhance the knowledge about dysphagia over the survivorship period. Finally, this study reports solely on patient-reported swallowing dysfunction instead of using the gold standard method for evaluating dysphagia (i.e., videofluoroscopy), which could have helped us to explore objectively how patients are impacted by their problems [1] and link it to subjective outcomes.

Despite the described limitations, our results provide evidence of a combination of symptoms that may be considered to better address the dysphagia perceived by sHNC with specific treatments developed by health care professionals. Due to the existence of multiple types of HNC and different matching treatments and/or supportive treatments (such as tracheal intubation), it is crucial to find specific management strategies that can more effectively regulate and control symptoms to improve sHNC’ QoL through the start-up of dysphagia prevention programs that would benefit patients and caregivers. This preliminary study may help researchers and clinicians to better understand the clusters of symptoms that occur during the survivorship phase in HNC.

## Conclusion

Dysphagia affected the great majority (85.5%) of sHNC in the present study. Moreover, coughing, reduced MMO and sleep disorders may act as predictors contributing to the development of dysphagia. Our results emphasize the importance of an early and proper identification of the symptoms as well as an adequate treatment strategy to address the cluster of symptoms that sHNC undergo.

### Supplementary Information

Below is the link to the electronic supplementary material.

Supplementary file1 (PDF 144 KB)
